# Divergent adaptation promotes reproductive isolation among experimental populations of the filamentous fungus *Neurospora*

**DOI:** 10.1186/1471-2148-8-35

**Published:** 2008-01-31

**Authors:** Jeremy R Dettman, James B Anderson, Linda M Kohn

**Affiliations:** 1 Department of Ecology and Evolutionary Biology, University of Toronto, Mississauga, ON, L5L 1C6, Canada

## Abstract

**Background:**

An open, focal issue in evolutionary biology is how reproductive isolation and speciation are initiated; elucidation of mechanisms with empirical evidence has lagged behind theory. Under ecological speciation, reproductive isolation between populations is predicted to evolve incidentally as a by-product of adaptation to divergent environments. The increased genetic diversity associated with interspecific hybridization has also been theorized to promote the development of reproductive isolation among independent populations. Using the fungal model *Neurospora*, we founded experimental lineages from both intra- and interspecific crosses, and evolved them in one of two sub-optimal, selective environments. We then measured the influence that initial genetic diversity and the direction of selection (parallel versus divergent) had on the evolution of reproductive isolation.

**Results:**

When assayed in the selective environment in which they were evolved, lineages typically had greater asexual fitness than the progenitors and the lineages that were evolved in the alternate, selective environment. Assays for reproductive isolation showed that matings between lineages that were adapted to the same environment had greater sexual reproductive success than matings between lineages that were adapted to different environments. Evidence of this differential reproductive success was observed at two stages of the sexual cycle. For one of the two observed incompatibility phenotypes, results from genetic analyses were consistent with a two-locus, two-allele model with asymmetric (gender-specific), antagonistic epistasis. The effects of divergent adaptation on reproductive isolation were more pronounced for populations with greater initial genetic variation.

**Conclusion:**

Divergent selection resulted in divergent adaptation and environmental specialization, consistent with fixation of different alleles in different environments. When brought together by mating, these alleles interacted negatively and had detrimental effects on sexual reproductive success, in agreement with the Dobzhansky-Muller model of genetic incompatibilities. As predicted by ecological speciation, greater reproductive isolation was observed among divergent-adapted lineages than among parallel-adapted lineages. These results support that, given adequate standing genetic variation, divergent adaptation can indirectly cause the evolution of reproductive isolation, and eventually lead to speciation.

## Background

Adaptation to different environments is postulated to play a large role in speciation and adaptive radiation [1]. With ecological speciation, reproductive isolation between populations evolves incidentally as a by-product of adaptation to divergent environments [2,3]. There are three potential sources of this reproductive isolation. First, the traits that are under divergent selection may indirectly reduce reproductive success by altering mate choice. Second, ecological mechanisms may contribute directly to reproductive isolation among species. If two species are specialized to different environments, interspecific hybrids may have reduced fitness in both environments because they lack the full complement of adaptive factors possessed by each of the parental species and exhibit a maladaptive intermediate phenotype. Third, divergent adaptation may promote reproductive isolation through genetic incompatibilities, as predicted by the Dobzhansky-Muller model [4,5]. When populations adapt to divergent environments, selection promotes sorting and fixation of different alleles in the alternate populations. Subsequent hybridization creates novel combinations of alleles that have not been tested and approved by selection, creating the possibility of negative, genetic interactions [6]. Under the Dobzhansky-Muller model, this antagonistic epistasis between incompatible alleles at interacting loci causes reductions in reproductive success. Given enough time, two completely separated populations will inevitably develop genetic incompatibilities due to mutation and genetic drift. Does divergent adaptation accelerate or increase the likelihood of the evolution of genetic incompatibilities, and thus speciation?

The amount of standing genetic variation in the ancestral population before speciation has a major effect on the evolution of reproductive isolation. Low-diversity populations may be restricted to adaptive peaks that are proximal to that of their conspecific ancestors, thereby limiting their ability to explore alternative adaptive peaks. High-diversity populations created by interspecific hybridization may possess greater segregational variance, with the potential for significant negative or positive epistasis among genetic factors. Positive epistasis may allow hybrid populations to access higher-fitness peaks elsewhere on the fitness landscape, thereby facilitating greater and more rapid evolutionary responses to novel environments. Furthermore, the level of potential divergence as a result of differential sorting between lineages increases with genetic variance. Hybrid speciation theory therefore suggests that the greater genetic variation associated with hybrid populations may increase the likelihood and rate of ecological speciation [7].

To test these key predictions of ecological and hybrid speciation, we developed an experimental evolution system using the filamentous fungus *Neurospora*. Several attributes make *Neurospora *ideal for this study. Like other microbes, it is amenable to experimental evolution because of short generation time, and ease of indeterminate propagation and archival storage [8]. The amount of genetic variation in artificial populations can be controlled by crossing parents of known relatedness. Also, the role of ecological specialization in promoting species divergence in this genus finds support in phylogenetic reconstructions [9].

Most importantly, *Neurospora *has a well-characterized sexual system with identifiable events associated with mating and sexual development [10,11]. Defects that cause reproductive isolation can be easily recognized at numerous stages in the sexual cycle [12-14]. In outbreeding Neurospora, mating occurs between haploid individuals of opposite mating type (*mat A *or *mat a*). Each self-sterile individual produces receptive "female" mating structures (protoperithecia) and fertilizing "male" propagules (conidia). Successfully fertilized protoperithecium enlarge rapidly and develop into mature fruiting bodies called perithecia. At this point, prezygotic isolation can be assessed by perithecial production. Karyogamy, meiosis, and mitosis occur to produce the haploid progeny (ascospores) which are then forcibly ejected from the perithecium. At this point, postzygotic isolation can be assessed by progeny viability.

Most experimental-evolution studies of reproductive isolation have focused on premating isolation and mating preference in *Drosophila *[3,15,16]. This form of isolation does not play an important role in speciation in fungi and plants because these organisms do not have "behaviour" in the classical, zoological sense. Here we focus on the incompatibilities that occur after mating (gamete transfer), which are central to reproductive isolation in all eukaryotic organisms.

In a recent study of the unicellular yeast, *Saccharomyces cerevisiae*, divergent adaptation resulted in both extrinsic ecological isolation and intrinsic genetic incompatibilities [17]. It is not known, however, if microbes with different levels of biological complexity respond to divergent, ecological selection in similar ways [18]. *Neurospora *has a filamentous rather than unicellular growth form, with more complex mating and sexual development than yeast. Here we study speciation with a similar experimental approach to that of ref. 17 but use a multicellular microbial system. In brief, we evolved replicate lineages of *Neurospora *in one of two sub-optimal, selective environments. The asexual fitness of lineages was then measured to test for adaptation to the selective environment and trade-offs in the alternate environment. To test for the development of reproductive isolation (both prezygotic and postzygotic), the success of matings between lineages was assessed using two different measures. By varying the amount of standing genetic (and phenotypic) diversity in the founding populations, we tested if increased variation caused by hybridization facilitates adaptation and the evolution of reproductive incompatibilities. To test if divergent adaptation increases the likelihood of the development of reproductive isolation, the effects of chance and divergent selection were differentiated by comparing the reproductive success between parallel-adapted lineages and divergent-adapted lineages.

## Results

### Evolution regimen

**Figure 1:**
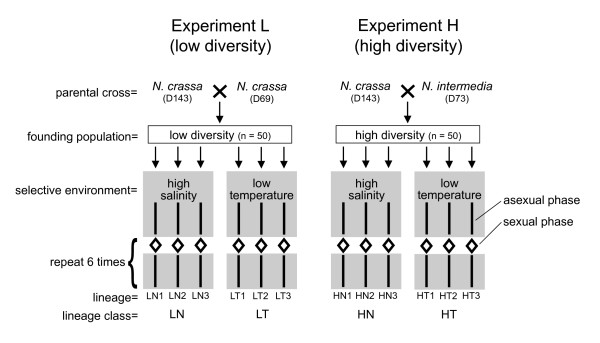
**Schematic of the evolution regimen for the experimental lineages of *Neurospora***. Founding populations were composed of 50 progeny (25 of each mating type) from one of two parental crosses. Lineages were exposed to one of two selective environments (grey background) during asexual propagation, which is indicated by solid lines. Diamonds indicate sexual cycles in which intra-lineage recombination occurred.

The founding populations for Experiment L (low diversity) and Experiment H (high diversity) were produced by an intraspecific and interspecific cross, respectively (Fig. 1).

The average sequence divergence between the parental individuals in Exp. H was 4.87%, nearly three times greater than that in Exp. L (1.63%; ref. 9). Replicate lineages of *Neurospora *were experimentally evolved for seven cycles of propagation, with each cycle consisting of a sexual phase followed by an asexual phase (Fig. 1). Divergent selection was applied during asexual reproduction by exposing the lineages to one of two sub-optimal, selective environments: high salinity or low temperature. For Exp. L, lineages evolved in high salinity or low temperature were named LN or LT, respectively. For Exp. H, lineages evolved in high salinity or low temperature were named HN or HT, respectively. In total, each experimental lineage underwent 7 meiotic generations and ~1500 mitotic generations.

### Adaptation

**Figure 2:**
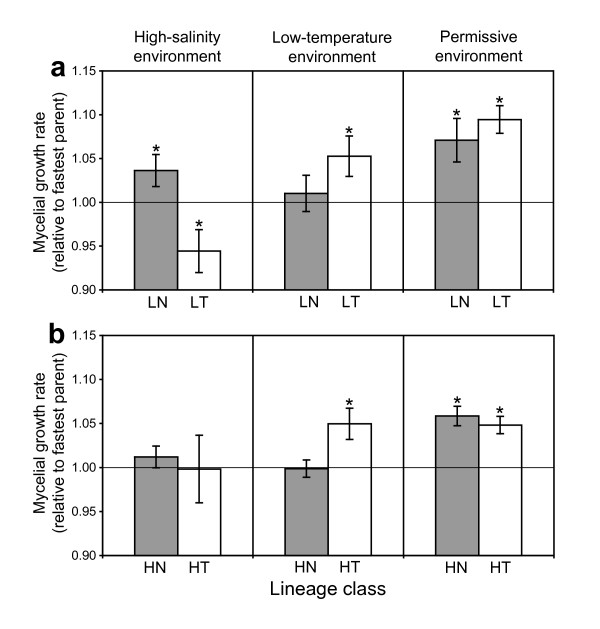
**Mycelial growth rates of evolved lineages from Experiment L (a) and Experiment H (b) at assay point 7**. Growth rates were normalized over the growth rate of the fastest parental progenitor in each of the three environments. Asterisks indicate means that were significantly different than 1.0, as determined by t-tests (see Methods). Errors bars are standard error (*n *= 6).

At the end of the evolution regimen, we tested for adaptation by assaying the linear, mycelial growth rate of evolved lineages in their respective selective environments (high salinity or low temperature). All four lineage-classes (LN, LT, HN, HT) had a significantly greater growth rate than the mid-parent mean in the selective environment (one tailed, *df *= 5, *p *≤ 0.01 in each case), with an average fitness advantage of 12.8% (± 1.2 SE). All four lineage classes also had mean growth rates that were greater than the fastest parental progenitor in the selective environment (Fig. 2), significantly for three lineage classes (one tailed, *df *= 5; LN, *p *= 0.05; LT, *p *= 0.04; HN, *p *= 0.19; HT, *p *= 0.02). On average, lineages gained a 3.8% (± 0.9 SE) fitness advantage over the fastest parental progenitor.

To test for trade-offs, growth rate was assayed in the alternate, selective environment. The evolved lineages displayed an average fitness reduction of 1.2% (± 1.3 SE), relative to fastest ancestor. Only the LT lineage class had a significantly reduced growth rate in high salinity (Fig. 2; one tailed, *df *= 5, *p *= 0.04). In the permissive environment (Fig. 2), each lineage class had a significantly greater growth rate than the fastest parent (two tailed, *df *= 5, *p *≤ 0.04 in each case), with an average fitness gain of 6.8% (± 0.8 SE).

In nature, the growth rate of evolved lineages in relation to each other, rather than in relation to the ancestor, is what determines competitive fitness. When assayed in the high-salinity environment, high-salinity-evolved lineages had a greater mean growth rate than low-temperature-evolved lineages (one tailed, *df *= 5; LN > LT, *p *< 0.007; HN > HT, *p *= 0.37). Similarly, low-temperature-evolved lineages had a greater growth rate than high-salinity-evolved lineages when assayed in the low-temperature environment (LT > LN, *p *= 0.10; HT > HN, *p *= 0.02). When modeled with ANOVA, the interaction between assay environment and selective environment was significant for Exp. L (*df *= 1, *p *= 0.0038), but not for Exp. H (*p *= 0.19). Despite these mixed significance levels, the overall pattern suggested a negative correlation of fitness gains across environments.

### Sexual reproductive success – progeny viability

To monitor the effects of environmental selection on the sexual reproductive success among lineages, we assayed progeny viability during the course of evolution. Assay points 1–7 were designated at the end of each of the seven sexual-asexual propagation cycles. Progeny viability assays were performed by crossing each lineage with the five other evolved lineages and measuring ascospore germination under permissive conditions. Given the reciprocity of matings regarding gender (male/female) and mating type (*mat A*/*mat a*), 4 different matings were performed for each of the 15 pairwise combinations of lineages within each experiment. Thus, each assay comprised 60 matings and the scoring of approximately 10,000 ascospores.

Evolution in sub-optimal conditions initially reduced progeny viability, however, regular sexual cycles subjected the experimental lineages to selection for greater reproductive success. As a response, progeny viabilities increased an average of 0.06 (± 0.06 SE) and 0.03 (± 0.02 SE) per successive assay point for Exp. L and H, respectively. Over the course of the evolution regimen, progeny viabilities had nearly recovered to the values observed for the original, parental crosses (Exp. L parental cross = 0.78; Exp. H parental cross = 0.17).

**Figure 3:**
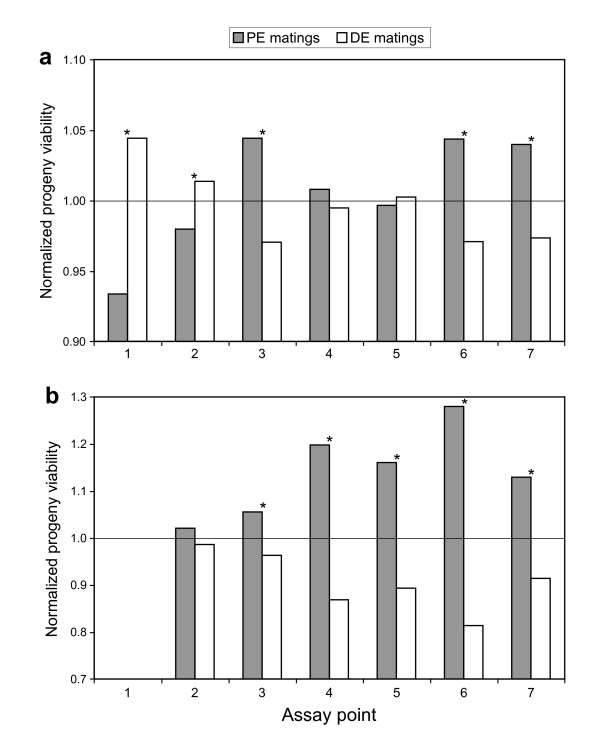
**Normalized progeny viability in the permissive environment for all matings in Experiment L (a) and Experiment H (b)**. Matings between lineages evolved in parallel environments (PE matings, *n *= 24) or divergent environments (DE matings, *n *= 36) are in grey or white, respectively. Mean progeny viability for each class was normalized over the grand mean for each assay point. Asterisks indicate significance differences between mating classes, as determined by Fisher exact tests (two-tailed) using ascospore germination/non-germination frequencies. Mean *n *per assay > 10,000 ascospores scored.

To test for the effects of parallel and divergent selection on reproductive success, we compared the progeny viability of matings between lineages evolved in parallel selective environments (PE matings) versus those evolved in divergent selective environments (DE matings). To account for variation in overall means, progeny viability was normalized over the grand mean for each assay point, allowing direct comparison of different assays. In Exp. L, progeny viability of PE matings was greater than that for DE matings (Fig. 3a) for some but not all assay points. In the early assays, significant differences were actually in the opposite direction than predicted by ecological speciation. However, this pattern was reversed as evolution progressed: the last two assay points showed significant differences (PE > DE, Fig. 3). In Exp. H, the results were clearly consistent with predictions: PE matings were significantly more successful than DE matings for assays points 3 through 7 (Fig. 3b).

We then modeled the effects of mating type and selective environment on progeny viability in Exp. L and H. The ANOVA included only assay points 6 and 7, the most derived states of the lineages (Table 1). Because each lineage could act as a female or a male in different matings, we analyzed these two gender roles independently. The mating type and selective environment of males were inconsequential to progeny viability (*p *≥ 0.24 in each case). In contrast, progeny viability was significantly affected by the selective environment of the female (*p *≤ 0.004 in both cases). Subsequent analyses therefore were performed separately on each lineage class as female.

**Table 1:** Summary of analysis of variance for progeny viability.

		Assay Point 6		Assay Point 7	
Experiment	Variation term	*F*	*p*	*F*	*p*
L (low diversity)	Female mating-type	0.794	0.377	0.687	0.411
	Female environment	3.235	0.078	9.223	**0.004**
	Interaction	1.659	0.203	0.036	0.851
	
	Male mating-type	0.769	0.384	0.602	0.441
	Male environment	0.789	0.378	0.616	0.436
	Interaction	2.140	0.149	0.559	0.458

H (high diversity)	Female mating-type	0.151	0.699	0.742	0.393
	Female environment	16.772	**<0.0001**	17.292	**<0.0001**
	Interaction	0.037	0.848	0.753	0.389
	
	Male mating-type	0.117	0.734	0.589	0.446
	Male environment	0.140	0.710	1.357	0.249
	Interaction	0.283	0.597	1.348	0.251

Sources of variation were mating type (*mat A *versus *mat a*), selective environment (high salinity versus low temperature), and their interaction. Significant *p *values are indicated in bold. Female and male gender roles were analyzed separately. In all cases, the *df *= 1 for each effect term, and *df *= 56 for the error term. All data were arcsine square-root transformed.

**Figure 4:**
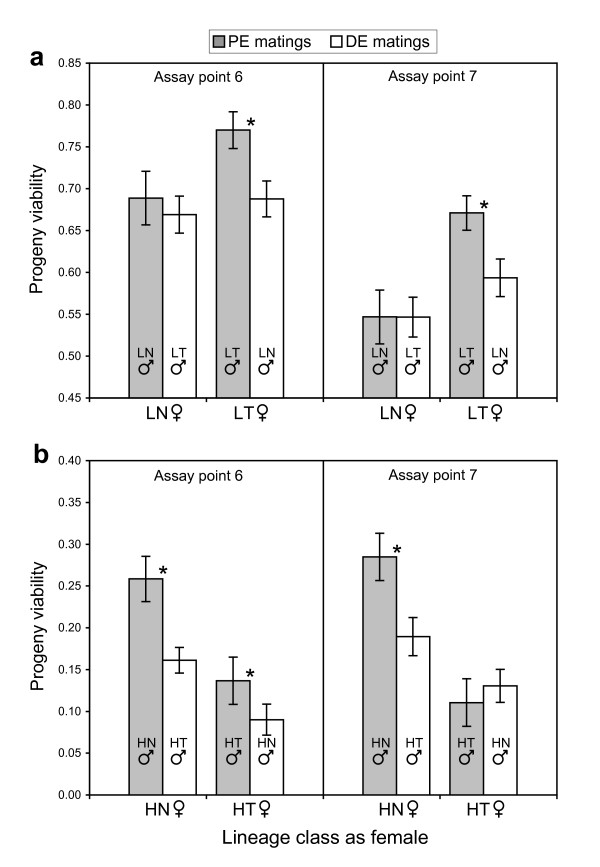
**Progeny viability in the permissive environment for matings in Experiment L (a) and Experiment H (b)**. Matings in which the female lineage was fertilized by males evolved in the parallel environment (PE matings, *n *= 12) or the divergent environment (DE matings, *n *= 18), are in grey or white, respectively. For clarity, the paternal lineage-classes are also shown. Asterisks indicate significance differences between mating classes, as determined by Fisher exact tests (two-tailed) using ascospore germination/non-germination frequencies. Mean *n *per maternal lineage-class > 3360 ascospores scored. Errors bars are standard error.

In general, progeny viability was greater when females were fertilized by males evolved in the parallel environment than when fertilized by males evolved in the divergent environment. These differences were significant at both assay points for one of the two lineage classes in each experiment: the LT and HN lineage classes in Exp. L and H, respectively (Fig. 4). These patterns were shared among lineages within lineage classes: PE > DE matings for all three LT and HN lineages (data not shown). In addition, PE matings were significantly more successful than DE matings for 5 of the assay points along the course of evolution (data not shown, Fisher exact tests; LT, *p *≤ 0.0004 in each case; HN, *p *< 0.0001 in each case), demonstrating a clear trend through time.

### Progeny viability in selective environments

**Figure 5:**
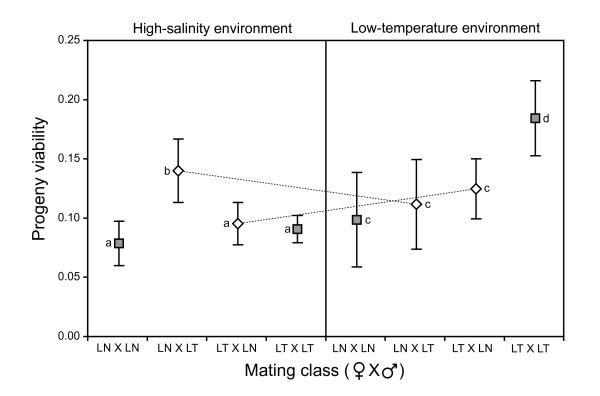
**Progeny viability assays performed in the two selective environments, using a subset of matings in Experiment L at assay point 7**. The PE matings are indicated by grey squares and are grouped by which selective environment both lineages classes were adapted to. The DE matings are indicated by white diamonds and are grouped by which lineage class acted as the female. For DE matings, dashed lines join the same mating class in the two selective environments. Means not connected by the same letter are significantly different from each other, as determined by Fisher Exact tests for each environment separately. Mean *n *per mating class > 668 ascospores scored per environment. Errors bars are standard error.

All previous assays of progeny viability were performed under permissive conditions. To examine the effects of sub-optimal conditions, we also scored progeny viability in high-salinity and low-temperature environments (30 matings, Exp. L). If environment-specific adaptation in the asexual phase had a correlated effect on ascospore germination, a U-shaped distribution of means across the two panels in Figure 5 is expected (based on the partners comprising each mating class). In the low-temperature environment, the rank order of progeny viability for the four mating classes was consistent with such a distribution. In the high-salinity environment, LN × LN matings had unexpectedly low viabilities and did not display a correlated effect: this represented the only case where the observed rank order did not match expectations. Owing to the small sample size, few significant differences were detected but notable trends did exist. In low temperature, LT × LT had significantly greater progeny viability than the other mating classes. For DE matings (Fig. 5), progeny viability was similar across both selective environments. When the assay environment matched the environment in which the female lineage had evolved, progeny viability was greater (interaction between selective environment and assay environment). This pattern again demonstrates environment-specific adaptation and the importance of the maternal contribution to progeny.

### Sexual reproductive success – perithecial production

Progeny viability measures postzygotic isolation and reflects the quality of progeny produced at the completion of sexual reproduction. Perithecial production measures prezygotic isolation and reflects the success of fertilization events. Perithecial number also influences the quantity of progeny produced, assuming that more perithecia produce more progeny.

**Figure 6:**
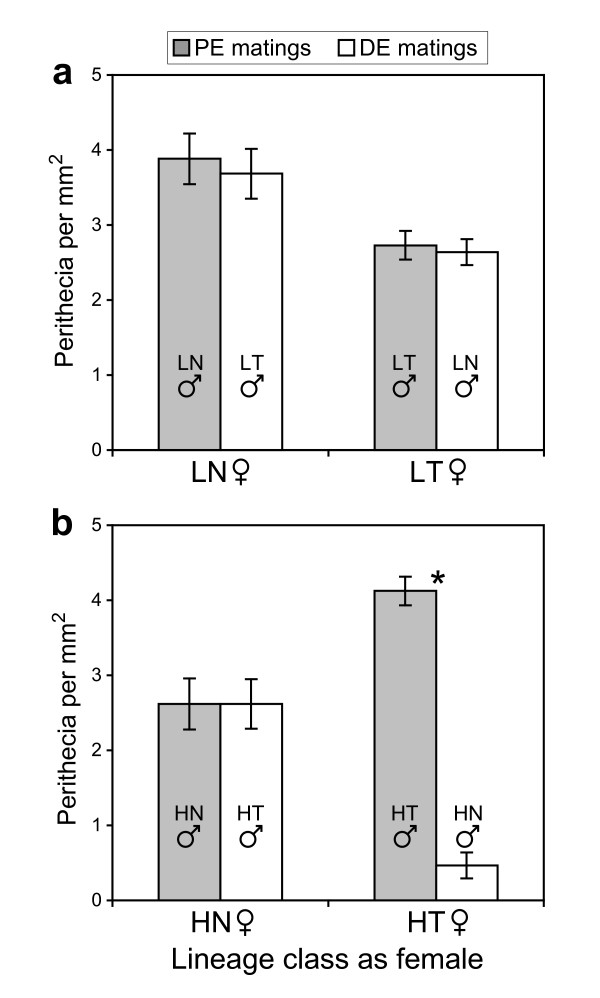
**Perithecial production for matings in Experiment L (a) and Experiment H (b) at assay point 7**. Matings in which the female lineage was fertilized by males evolved in the parallel environment (PE matings, *n *= 12) or the divergent environment (DE matings, *n *= 18), are in grey or white, respectively. For clarity, the paternal lineage-classes are also shown. Full assays were performed once and four times for Experiment L and H, respectively. Asterisks indicate significance differences between mating classes, as determined by two-tailed t-tests. Errors bars are standard error.

Perithecial production was measured at assay point 7 and evidence for reproductive isolation was observed in Exp. H but not Exp. L. For Exp. L, no significant differences in perithecial production were detected between PE and DE matings (Fig. 6a; *p *> 0.67 for each lineage class as female). For Exp. H, PE matings produced significantly greater numbers of perithecia than DE matings, but only when HT lineages acted as the female (Fig. 6b; *p *< 0.0001). In HT(female symbol ♀) × HN(male symbol ♂) matings, perithecial production was greatly reduced compared to wild-type levels (Fig. 6b). The protoperithecia responded to fertilization by expanding and darkening slightly, the typical response seen in wild-type matings. However, the vast majority of perithecia aborted at this stage of development and never matured into the larger, black perithecia. In the reciprocal HN(female symbol ♀) × HT(male symbol ♂) matings, an incompatibility phenotype was not observed: nearly all perithecia developed to maturity as normal. All combinations of HT and HN lineages shared this pattern of asymmetrical reproductive isolation (data not shown). The two types of matings differed only by which lineage class functioned as the female and male partner, demonstrating that the incompatibility was expressed in a gender-role-specific fashion.

### Genetic basis of incompatibility phenotype

The genetic basis of the interactions causing the reductions in perithecial production was investigated further. Sixty f_1 _progeny were isolated from crosses between HT2 and HN2 lineages. These progeny were mated back to the HT2 and HN2 parents, in both gender roles, and perithecial production was assayed.

When the f_1 _progeny were grown as females and fertilized by the HT2 and HN2 males, perithecial production values formed a bimodal distribution (not shown). The discriminatory phenotype, defined as a female producing >3 times more perithecia when fertilized by HT2 males than when fertilized by HN2 males, segregated 30:30 (χ^2^, *p *= 1.0). These two lines of evidence suggested that the phenotype was controlled by a single, Mendelian locus. This locus was named *dfe *for "d*iscriminatory *fe*male*", and the alleles that confer discrimination or non-discrimination were named *dfe*^*d *^and *dfe*^*n*^, respectively.

When these progeny were grown as males and used to fertilize discriminatory HT2 females, perithecial production values again formed a bimodal distribution (not shown). The discriminated phenotype, defined as the inability of a male to activate an HT2 female to produce >1 perithecia/mm^2^, was observed in 26 of the 60 f_1 _progeny (χ^2^, *p *= 0.30). This segregating locus, which controls the "d*iscriminated *ma*le*" phenotype, was named *dma *and the alleles that confer discrimination or non-discrimination were named *dma*^*d *^and *dma*^*n*^, respectively.

The inferred genotypes of the HT2 and HN2 parents were *dfe*^*d *^*dma*^*n *^and *dfe*^*n *^*dma*^*d*^, respectively. Among the 60 f_1 _progeny, we observed 34 parental (19 *dfe*^*d *^*dma*^*n*^; 15 *dfe*^*n *^*dma*^*d*^) and 26 recombinant (11 *dfe*^*d *^*dma*^*d*^; 15 *dfe*^*n *^*dma*^*n*^) genotypes, providing evidence that the two loci were genetically unlinked (χ^2^, *p *= 0.55). We selected 8 f_1 _progeny (4 of each mating type, 2 of each two-locus genotype) and assayed perithecial production in the 32 possible matings among them. All observed phenotypes matched predictions based on the female × male genotype combinations: perithecial production was reduced only for matings in which the female was *dfe*^*d *^and the male was *dma*^*d *^(Table 2).

**Table 2:** Mean perithecial production for matings between all combinations of the 4 two-locus genotypes.

	Male genotype			
	
Female genotype	*dfe*^*d *^*dma*^*d*^	*dfe*^*d *^*dma*^*n*^	*dfe*^*n *^*dma*^*d*^	*dfe*^*n *^*dma*^*n*^
*dfe*^*d *^*dma*^*d*^	**0.25 **(0.05)	3.79 (0.20)	**0.27 **(0.05)	3.58 (0.36)
*dfe*^*d *^*dma*^*n*^	**0.28 **(0.07)	3.09 (0.50)	**0.31 **(0.06)	3.58 (0.31)
*dfe*^*n *^*dma*^*d*^	4.10 (0.18)	3.27 (0.17)	3.69 (0.20)	3.46 (0.19)
*dfe*^*n *^*dma*^*n*^	4.33 (0.41)	4.00 (0.71)	3.73 (0.85)	4.30 (0.37)

Each cell of the matrix contains the mean number of perithecia produced per mm^2 ^(± standard error). *n *= 4 (2 *mat A*/*mat a *reciprocal matings, 2 replicates each). Bold values indicate genotype combinations that displayed the incompatibility phenotype.

We were able to reconstitute the original, parental HT2 and HN2 genotypes, and associated phenotypes, by crossing recombinant f_1 _genotypes (*dfe*^*n *^*dma*^*n *^× *dfe*^*d *^*dma*^*d*^). Among 24 f_2 _progeny, we obtained 13 parental (9 *dfe*^*d *^*dma*^*d*^; 4 *dfe*^*n *^*dma*^*n*^) and 11 recombinant (6 *dfe*^*d *^*dma*^*n*^; 5 *dfe*^*n *^*dma*^*d*^) genotypes (χ^2^, *p *= 0.51). Again, the two alleles at both loci segregated evenly in these f_2 _progeny (χ^2^, *p *> 0.22 in both cases).

## Discussion

The founding populations of *Neurospora *were composed of multiple individuals with standing genetic variation. As evolution progressed, the genetic composition of these initially identical lineages could change by the sorting of pre-existing and new alleles, or by the creation of novel allele combinations through recombination. How these processes interact with the different types and strengths of selective pressures during the evolution regimen ultimately determines the phenotype of the end-point lineages. In this study, we investigated the effects of these processes on adaptive increases in asexual fitness and the evolution of reproductive isolation.

The best evidence for adaptation is when the evolved lineages have greater fitness than their most-fit ancestor (Fig. 2). Although specialization to one environment may be associated with fitness reductions in other environments [19,20], we found only limited evidence for such fitness trade-offs (Fig. 2). These results are fully consistent with the recent experimental study of speciation in yeast [17]. Taken together, the mycelial growth rate data demonstrate that the negative correlation of fitness gains in the two selective environments was a result of divergent environmental selection. Surprisingly, divergent selection also caused a correlated response of an increase in general growth rate in the permissive environment (Fig. 2). Furthermore, environmental adaptation in the asexual phase had a correlated effect on ascospore germination in those selective environments (Fig. 5). This pattern is expected if ascospore germination is considered to be the first step in mycelial growth.

Compared to previous experimental studies with fungi [17,21-24], the levels of environmental adaptation we observed here were unexpectedly low. We used mycelial growth rate as a proxy for fitness because it was the most obvious trait under selection; the mycelial sector with the fastest growth rate was transferred as the founder for the next culture tube. Other asexual traits, such as biomass accumulation, may have also been under selection, but were not measured. In addition, selection during sexual cycles may have counteracted selection during the asexual phase, causing a reduction in the net asexual fitness gains [25-27].

Another factor potentially impeding adaptation is the coenocytic growth form of this filamentous fungus. Hyphae of *Neurospora *are densely packed with nuclei that are capable of migrating between hyphal compartments. The interconnectedness of the mycelial network may have a homogenizing effect, interfering with competition among genetically different nuclei. Individual nuclei have, at best, only partial control of the phenotype and are not fully exposed to selection. Propagation of lineages by conidia, in which individual nuclei are more exposed to selection than in hyphal tips, may have allowed for more efficient competition and a greater adaptive response [28]. Further comparative, experimental studies are needed to determine the relationship between growth form and adaptive potential of filamentous fungi.

When ecological speciation via divergent adaptation occurs, there are two possible underlying causes of sexual reproductive isolation. First, direct selection on the sexual phase may cause specialization to a particular environment, such that sexual reproduction is disrupted when not within that environment. In this case, reductions in reproductive success are caused by interactions between adapted genotypes and the environment ("ecological isolation"). Second, divergent selection during the asexual phase may cause the accumulation of negatively interacting loci, which consequently have indirect effects on the sexual phase. In this case, reductions in reproductive success are caused by antagonistic epistasis between genetic factors in the adapted genomes ("genetic isolation").

To discriminate the direct effects of divergent adaptation during the sexual phase from the indirect effects of divergent adaptation during the asexual phase, these two selective pressures were experimentally decoupled. The *Neurospora *populations were exposed to selective, divergent environments only during the asexual phase. Most organisms spend the majority of their life in the asexual phase, so adaptive evolutionary responses to environmental selection typically occur in this phase. At no point in our evolution regimen was reproductive isolation the target of selection. The sexual phase was performed under identical, permissive, conditions for all lineages. Thus, any differential patterns of sexual reproductive success could be attributed to the indirect effects of divergent selection on non-sexual traits that had overpowered the unifying effects of parallel selection on the sexual phase.

Our experimental design also allowed us to identify genetic isolation. When sexual reproductive success was assayed in the permissive environment, any patterns of differential reproductive success could be attributed solely to genetic isolation, and not to ecological isolation. One may argue that, in nature, genetic isolation is far more important than ecological isolation because the latter is, by definition, dependent upon the environment. Incompatibilities caused by intrinsic genetic interactions may be permanent despite changing environments.

We could determine if the development of reproductive isolation was caused by random effects of chance or by deterministic effects of divergent selection. If the evolution of reproductive incompatibilities was random, these phenotypes should not be correlated with the selective environments: parallel and divergent selection should have similar effects on reproductive isolation. Thus, divergent selection can be implicated as the causal factor only when greater reproductive isolation is observed between divergent-adapted lineages than between parallel-adapted lineages (Figs. 3, 4, 6).

In general, the conclusion that divergent adaptation promotes the development of reproductive isolation was supported by results from progeny viability assays, particularly from the later stages of the experiments (Fig. 3). Differential progeny viability that was consistent with ecological speciation was observed in almost all comparisons made at the level of lineage class as female (Fig. 4). The two lineage classes (LT and HN) that drove the trend were evolved in different environments, and were derived from founding populations with different levels and sources of genetic variation. Thus, differential progeny viability was not dependent upon a particular selective environment, or particular genetic components possessed by the progenitors. Rather, divergent selection in divergent environments caused the patterns of differential reproductive success.

Perithecial production assays for Exp. H, but not Exp. L, were consistent with divergent adaptation promoting the development of reproductive isolation (Fig. 6). For Exp. H, females produced more perithecia when fertilized by parallel-adapted males than when fertilized by divergently-adapted males. When HT lineages functioned as the female, the discrepancy was quite dramatic: PE matings produced, on average, 9.39 times more perithecia than DE matings.

The incompatibility phenotype and its inheritance pattern support a two-locus, two-allele model with asymmetric, antagonistic epistasis. The two loci, *dfe *and *dma*, interact in a gender-role-specific fashion to modulate the progression through the sexual cycle. Antagonistic interactions between the *dfe*^*d *^and the *dma*^*d *^alleles cause major reductions in perithecial production, but only when the female possesses the *dfe*^*d *^allele and the male possesses the *dma*^*d *^allele. The reciprocal pairing, and all other allele pairings, do not result in the incompatibility phenotype. This model is fully compatible with the Dobzhansky-Muller model.

Reductions in reproductive success are compounded over the successive stages of a single life cycle. When perithecial production and progeny viability are combined, PE matings have a 7.64-fold advantage over DE matings for Exp. H. For Exp. L, in which the degree of differential reproductive success was much lower, PE matings have only a 1.13-fold (13%) advantage over DE matings. Although this is not a large difference, one must consider that these effects are compounded over successive generations, allowing minor disparities in reproductive success to have substantial consequences on gene flow between populations.

We are aware of only six other experimental evolution studies in which the effects of divergent selection and genetic drift could be separated. Recent work with experimental populations of yeast clearly demonstrated that divergent adaptation resulted in significantly greater reproductive isolation than parallel adaptation [17]. The other five studies all used Drosophila as the experimental system, but obtained mixed results. In four studies [29-32], divergent selection had greater effects than parallel selection. In another study [33], however, the predictions of ecological speciation were not supported.

This study revealed the strong influence that the maternal partner has on the success of a mating. These results are explicable for *Neurospora *because the female bears nearly the whole burden of sexual reproduction. The maternal partner forms complex structures for mating, actively senses the presence of a male, and produces most of the tissue comprising a mature perithecium. In contrast, the only role of the male is to passively donate nuclei to the female.

Increased genetic variance caused by interspecific hybridization allows for both negative and positive epistasis, thus expanding the fitness distribution in both directions. The Dobzhansky-Muller model focuses on negative interactions between heterospecific genomes that may cause less fit genotypes. Hybrid speciation theory focuses on positive interactions (and transgressive segregation) that may create more fit genotypes. In this sense, the two theories are opposite sides of the same coin. Here we tested whether the potential benefits of positive epistasis could overpower the potential detriments of negative epistasis, regarding the overall fitness of lineages and their ability to adapt; the results of our experiment suggest they cannot. Even though the multiple rounds of sex and recombination should have allowed the separation and purging of negatively interacting factors, lineages from the high-diversity Exp. H did not adapt to the selective environments more than the lineages from the low-diversity Exp. L. When mycelial growth rates in all three environments are considered (Fig. 2), it appears that Exp. L lineages actually had greater responses (both direct and correlated) to selection than Exp. H lineages.

Results from progeny viability and perithecial production assays supported predictions from hybrid speciation theory: differential reproductive success was more common and more striking in Exp. H than in Exp. L (Figs. 3, 4, 6). Exp. H was initiated from a cross between two species, *N. crassa *and *N. intermedia*, with nearly complete reproduction isolation [12-14]. The founding population likely contained considerable genetic variance for reproductive success. We hypothesize that, as variation sorted out during the course of evolution, lineages became fixed for particular combinations of compatible genomic regions. Parallel adaptation during the asexual phase promoted the fixation of similar genomic regions. Divergent adaptation promoted the fixation of dissimilar genomic regions, thereby increasing the likelihood of genetic incompatibilities among lineages.

Identifying the genomic regions and, ultimately, the genes that interact to produce the incompatibility phenotypes is critical to understanding the causes of reproductive isolation. Further research should investigate the genomic composition of the evolved lineages, and its correlation with adaptation and incompatibility. Genomic regions consistently shared among parallel-selected lineages could point to the genes or mutations under selection and involved in adaptation. The most discrete differences in reproductive success were in perithecial production, so it might be possible to determine the location and characteristics of the two genetic factors (*dfe *and *dma*). At this point, it is unclear if these loci encode single genes or sets of tightly linked genes. By genetic manipulation and controlled crosses, one could determine precisely the nature and mechanism of the antagonistic interaction that causes the Dobzhansky-Muller incompatibility.

## Conclusion

This study demonstrated three important aspects of speciation in a developmentally complex eukaryotic microbe. First, divergent selection on a common, founding population resulted in divergent adaptation and environmental specialization. Evolved populations typically had higher fitness in their selective environment than in the alternate environment in which they had not evolved. This negative correlation of fitness across environments provides the phenotypic variation upon which ecological mechanisms of isolation can act. Second, divergent adaptation resulted in greater reproductive isolation than parallel adaptation. Evidence for differential reproductive success was observed at two stages of the sexual cycle: perithecial production and progeny viability. The genetic basis for one of the observed incompatibility phenotypes can be explained by a two-locus, two-allele model with asymmetric, antagonistic epistasis. Third, the effects of divergent adaptation on reproductive isolation were more pronounced for populations with greater initial genetic variation. Interspecific hybridization may produce novel variation that allows the populations to follow previously inaccessible evolutionary trajectories. Taken together, our results support the hypothesis that, given adequate standing genetic variation, divergent adaptation can indirectly cause the evolution of differential reproductive success, and eventually lead to speciation.

## Methods

### Strains

Two strains of *N. crassa *subgroup NcA (D143 [*mat A*, FGSC 8903] and D69 [*mat a*, FGSC 8829]) and one strain of *N. intermedia *subgroup NiA (D73 [*mat a*, FGSC 8833]) were used in this study. Species tester strains of *N. crassa *(FGSC 6682 [*mat A*] and FGSC 6683 [*mat a*]) were used for mating-type determination. Information on these strains can be obtained from refs. 9 and 14, or the Fungal Genetics Stock Center (FGSC [34]). Unless otherwise stated, strains were cultured on Vogel's Minimal (VM) medium (1× Vogel's salt solution, 1.5% sucrose, 1.5% agar).

### Crossing and progeny isolation

The *Neurospora *species used in this experiment are heterothallic (self-sterile). Each individual can act as a "female" or "male", depending upon how the individual was prepared for mating and how the cross was performed. Gender is determined by the direction of fertilization and nuclear transfer. The individual or population to act as the female was grown in 13 × 100 mm test tubes containing slanted Westergaard's Synthetic Crossing (SC) medium (1× SC salts, 1% sucrose, 2% agar) to promote the development of specialized, female mating structures (protoperithecia). The male was grown on VM medium to produce mitotic propagules (conidia). After 4 d, the female was fertilized with a dense suspension of conidia harvested from the male. Hair-like protrusions (trichogynes) from the protoperithecia sense and actively grow towards nearby males through chemotaxis. Successfully fertilized hyphae proliferate within the enlarging protoperithecium that develops into a mature fruiting body (perithecium). Within the perithecium, apical segments of the fertilized hyphae (asci) undergo karyogamy, meiosis, and one mitotic division, with each ascus yielding eight haploid progeny (ascospores). When mature, the asci forcibly eject the ascospores out of an apical opening (ostiole) in the perithecium: the ascospores adhere to the inside test tube wall. Ascospores were harvested 18 d after fertilization, then heat-shocked for 40 min at 60°C to activate the germination process. Ascospores were spread on colony-restricting sorbose medium (1× Vogel's salt solution, 2.0% sorbose, 0.05% dextrose, 2.0% agar) and allowed to germinated for 24 h. Viable colonies formed by individual ascospores were transferred to separate test tubes. The mating type of progeny was determined by spotting conidial suspensions on lawns of female-receptive *mat A *and *mat a *tester strains.

### Founding populations

To produce the low-diversity, founding populations for Experiment L, an intraspecific cross between *N. crassa *D143 and *N. crassa *D69 was performed. For Experiment H, an interspecific cross between *N. crassa *D143 and *N. intermedia *D73 was used produce the hybrid, high-diversity, founding populations. Note that *N. crassa *D143 was a parent common to both experiments. The sequence divergence between parental individuals was the mean uncorrected genetic distance from four independent loci [9]. For each experiment, 25 progeny of each mating type were isolated. The individuals in each mating-type class were grown to the point of conidiation, harvested, then mixed in approximately equal proportions in sterile distilled water. Other than the hybrid origin of the founding populations in Exp. H, all other protocols were the same for Exp. L and H.

### Asexual propagation

For Exp. L, the 25-genotype, *mat A *founding population was divided into two groups of three sub-lineages, designated LN1-A, LN2-A, LN3-A, and LT1-A, LT2-A, LT3-A. Similarly, the *mat a *founding population was divided into sub-lineages designated LN1-a, LN2-a, LN3-a, and LT1-a, LT2-a, LT3-a. Lineages consisted of paired sub-lineages of opposite mating type; for example, lineage LN1 consisted of sub-lineages LN1-A and LN1-a. The same was done for Exp. H, except the lineages were given HN and HT names (see Fig. 1). To initiate each of the 12 sub-lineages, a 50 μl aliquot of the founding population mixture, containing approximately 5 million conidia, was inoculated into the end of glass tubes (2.5 × 30 cm) filled horizontally with 20 ml of solid media (1.5% agar). Conidia typically contain 2–3 nuclei, so the inoculum represents between 10–15 million nuclei. The LN and HN lineages were propagated asexually in a high salinity environment (VM medium with 0.7 M NaCl), whereas the LT and HT lineages were propagated in low temperature environment (VM medium incubated at 12°C). The sub-lineages of opposite mating type were propagated separately to avoid the loss of one mating type by drift. When cultures neared the end of tubes, a 15 × 20 mm strip of the entire leading colony front was transferred to a new tube with fresh medium. Not only were hyphae growing on the surface of the medium, but significant amounts of submerged and aerial hyphae were present. Thus, we assumed that 100% of the 300 mm^2 ^of two-dimensional area was colonized. Given that the average hyphal strand is 12 μm wide, and using the calculated nuclear densities (see below), we estimated that our asexual propagation regimen transferred approximately 25 million nuclei between successive culture tubes.

Experimental lineages underwent a total of 450 cm of asexual growth. Determination of the number of mitotic generations the lineages had evolved is not straight-forward for *Neurospora*. First, the coenocytic mycelium has numerous nuclei per hyphal compartment, and nuclei can migrate freely between compartments. Second, mitosis is not synchronized across nuclei. Third, nuclear division is not restricted to the growing front of the colony. These characteristics make tracking the history of individual nuclei impossible. Despite these difficulties, we estimated the number of cell cycles by two methods. The first estimate relied on the correlation between cell cycle duration and mycelial extension rate. Given a cell cycle time of 1.5 h [35] and a growth rate of 2.0 mm/h at 22°C on VM medium, 16.67 cell cycles are completed for each 5 cm of growth. The second estimate relied on the number of nuclei per unit length of hyphal filament. *Neurospora *hyphae are densely nucleated, with an estimated 1000 nuclei per 1 mm of hyphae [36,37]. A single hyphal filament of 5 cm in length, which approximates the length of the actively growing colony front, would contain ~50,000 nuclei. Assuming a single initial nucleus, and that all nuclei remain mitotically active, 15–16 cell cycles would be needed to generate 50,000 nuclei and 5 cm of growth. Thus, the two methods gave similar results, and we estimate that 450 cm of asexual growth represents approximately 1500 mitotic generations. Our generation calculations (16.67 cell cycles in 5 cm) are very conservative compared to estimates from other filamentous fungi (e.g. ref 38; 115 cell cycles in 3 cm), so we may in fact be greatly underestimating the generation number. Moreover, our estimates do not include any of the mitotic generations that occurred during the sexual cycles and progeny isolation.

### Sexual cycles

Asexual evolution was interspersed with six cycles of sexual reproduction (Fig. 1), with an average of 68 cm of asexual growth between cycles. To maintain the genetic cohesiveness of lineages, genetic exchange was achieved by crossing each sub-lineage with its paired sub-lineage of opposite mating type. To reconstitute each sub-lineage, 20 progeny of the appropriate mating-type were isolated and mixed in approximately equal proportions in sterile distilled water. Further asexual evolution was re-initiated from approximately 5 million conidia in solution. Including the initial founding cross, lineages underwent a total of 7 sexual cycles.

### Mycelial growth rate

Cultures were inoculated near the perimeter of petri plates (100 × 15 mm) containing the appropriate medium. To allow cultures to acclimate to the assay environment and reach a steady-state growth rate, measurements were started only after the culture had grown approximately 2 cm or more. Rates were averaged over the entire assay period, the duration of which depended upon the environment. Growth rates were determined for each sub-lineage by averaging three replicated measurements. The permissive environment was VM medium at 22°C. Mycelial growth rates were measured at the end of the evolution regimen.

To test for adaptation, the growth rate of an evolved sub-lineage was divided by the growth rate of the fastest parental progenitor. The *N. crassa *D143 parent was used in all cases because it had a greater growth rate than the other parent, for both Exp. L and H, and in all environments. The statistical significance of deviations from 1.0 was determined by t-tests. Tests for adaptation (>1) or trade-offs (<1) were one-tailed, whereas tests for correlated responses in the permissive environment were two-tailed.

### Sexual reproductive success – progeny viability

Progeny viability assays were performed by mating sub-lineages from the same experiment. A culture from each sub-lineage was grown for 3 d on VM slants, then used to inoculate 5 SC slants for mating. The physiological states of cultures prior to mating were similar because all cultures were grown outside of the selective environments, under permissive conditions, for two transfers: first on VM slants, then on SC slants. These steps ensured that the stress of growing in the selective environments did not affect reproductive success or confound the assessment of genetic isolation. Progeny viability assays consisted of 60 matings: each of the 12 sub-lineages was fertilized by the 5 different sub-lineages of opposite mating type. Each pairing of two sub-lineages consisted of two reciprocal matings: one with the *mat A *partner as female, one with the *mat a *partner as female. This design allowed the assessment of female function to be separated from male function.

For each mating, heat-shocked ascospores were spotted on sorbose medium and allowed to germinate at 22°C for 24 h. Progeny viability was quantified as the proportion of ejected ascospores that germinated to produce hyphae. For the full assays in the permissive environment, we scored an average of 170 ascospores from each mating, resulting in an average of 10,168 ascospores per assay. The significance of differences between mating classes was determined from germination/non-germination frequencies using Fisher Exact tests. Matings were weighted in inverse proportion to the number of ascospores scored, such that each mating contributed equally to the statistic. To represent the variation among matings within classes, germination counts were converted to proportions and standard errors were calculated.

When progeny viability was scored in the selective environments, we used only the 30 matings in which the *mat A *partner was female. Analyses of full assays showed that the mating type of the female did not affect reproductive success. These assays were performed at assay point 7 for Exp. L.

### Sexual reproductive success – perithecial production

The female was inoculated and grown on SC plates for 4 d to develop receptive protoperithecia. A 2.5 μl aliquot of a conidial suspension made from the male was spotted onto the female. After 8 d, we counted the maximum number of perithecia in the field of view of a dissecting scope at 50× magnification (19.63 mm^2^), from which the number of perithecia per mm^2 ^was calculated. At this developmental stage, successfully fertilized protoperithecia have commenced sexual development, as indicated by a darkening of the perithecial wall and the formation of beaks or beak-like protrusions. Such fruiting bodies were classified as perithecia. Protoperithecia that had not commenced sexual development by day 8 never matured into perithecia, as verified by inspection of the same areas 7 d later.

The concentration of conidia in the fertilizing solution was great enough that all protoperithecia presumably had access to conidia. When a subsample of fertilizations was repeated with 1/10 and 1/100 dilutions of conidial solutions, the same number of perithecia were produced. Thus, females were over 100× saturated by males.

## Authors' contributions

The research was conceived and planned by all authors. JRD performed the experiments, analyzed the data, and wrote the manuscript. All authors contributed to editing the manuscript and have approved the final version.
